# Diffuse punctate calcifications involving the colonic region

**DOI:** 10.4103/0971-4065.41288

**Published:** 2008-01

**Authors:** K. Sampathkumar, Y. S. Sooraj, A. R. Mahaldar, R. Muthiah, R. Ajeshkumar

**Affiliations:** Department of Nephrology, Meenakshi Mission Hospital and Research Centre, Lake Area, Melur Road, Madurai, Tamil Nadu - 625 107, India

**Fig. 1:**
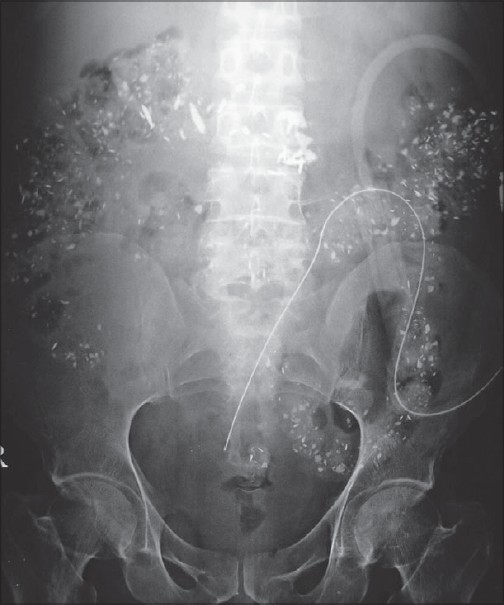
Plain X-ray abdomen (erect) of the patient

A 50-year-old gentleman diagnosed as having diabetic nephropathy, with end-stage renal disease (ESRD) one year ago, was advised continuous ambulatory peritoneal dialysis (CAPD) in view of repeated access failure in hemodialysis. His blood urea was 102 mg/dl; serum creatinine, 9.1 mg/dl; corrected serum calcium, 8.7 mg/dl and serum phosphorus level, 7.4 mg/dl. He underwent percutaneous Tenckhoff CAPD catheter insertion. A plain X-ray of the abdomen (erect) was obtained to confirm the position of the catheter [Fig. 1].

The X-ray showed diffuse punctate calcifications in the region of the colon along with the CAPD catheter *in-situ*.

Calcifications observed in flat plate films of the abdomen can be broadly divided into two types: those that are confined to a particular organ and those diffusely occupying the whole abdomen. In our patient, the distribution was more suggestive of punctate discrete calcifications in the region of the large intestine. He was on lanthanum carbonate 500 mg thrice a day for his hyperphosphatemia.

Recently, reports of incidentally discovered calcific shadows in the colonic region amongst patients on Lanthanum carbonate have been published.^1^,^2^ Lanthanum is a rare earth metal bearing atomic number 57, which is close to that of Barium (56). This phenomenon is important since misdiagnosis and endoscopic procedures can be avoided. This method can also be used to assess drug compliance.^2^
